# 43412 Exploring the link between allostatic load and mortality risk in U.S. Black men of different age groups

**DOI:** 10.1017/cts.2021.709

**Published:** 2021-03-30

**Authors:** Duane J. Wallace, Roland J. Thorpe

**Affiliations:** 1 Johns Hopkins University School of Medicine; 2 Johns Hopkins Bloomberg School of Public Health

## Abstract

ABSTRACT IMPACT: This research study will provide evidence for public policy, systemic changes, and other interventions to address the adverse impacts of prolonged stress exposure experienced by young Black men. OBJECTIVES/GOALS: Previous studies have demonstrated a strong association between allostatic load (i.e., stress-induced cumulative biological risk) and mortality in the Black American population. The aim of this study is to examine the association between allostatic load and mortality in Black men and to determine if the relation varies by age. METHODS/STUDY POPULATION: Data from the third National Health and Nutritional Examination Survey (NHANES III, 1988-1994), linked to the 2015 National Death Index Public Release File, will be used for Black male adults 18 years or older. Allostatic load score includes nine biomarkers: albumin, C-reactive protein, total cholesterol, high-density lipoprotein, hemoglobin A1C, waist-to-hip ratio, systolic blood pressure, diastolic blood pressure, and pulse rate. The number of variables for which the participant’s scores fall in the quartile of highest clinical risk are added together to create a summary score. Cox proportional-hazard analyses is employed to estimate the associations between allostatic load and all-cause mortality for the total sample and stratified by age, adjusting for selected characteristics. RESULTS/ANTICIPATED RESULTS: We hypothesize that the association of allostatic load with mortality will be greater among younger, compared to older, Black men. Young Black men (ages 25-44) are at particular risk of adverse impacts of chronic stress and allostatic load, due to their experience of chronic discrimination, systemic racism, racial battle fatigue, and mundane, extreme, environmental stress. Furthermore, the allostatic load-mortality association may be attenuated for older Black men due to a survival effect. DISCUSSION/SIGNIFICANCE OF FINDINGS: If the association between allostatic load score and mortality is stronger in young Black men, it would provide evidence for early identification of a group with high risk of premature mortality, and for public policy, systemic changes, and other interventions to address the adverse impacts of prolonged stress exposure experienced by young Black men.

